# Mitochondrial Targeted Endonuclease III DNA Repair Enzyme Protects against Ventilator Induced Lung Injury in Mice

**DOI:** 10.3390/ph7080894

**Published:** 2014-08-22

**Authors:** Masahiro Hashizume, Marc Mouner, Joshua M. Chouteau, Olena M. Gorodnya, Mykhaylo V. Ruchko, Glenn L. Wilson, Mark N. Gillespie, James C. Parker

**Affiliations:** 1 Department of Physiology, University of South Alabama, Mobile, AL 36688, USA; 2 Center for Lung Biology, University of South Alabama, Mobile, AL 36688, USA; 3 Department of Pharmacology, University of South Alabama, Mobile, AL 36688, USA; 4 Department of Cell Biology and Neuroscience, University of South Alabama, Mobile, AL 36688, USA

**Keywords:** endonuclease III, albumin spaces, MIP-2, IL-6, glutathione, pulmonary edema, vascular permeability

## Abstract

The mitochondrial targeted DNA repair enzyme, 8-oxoguanine DNA glycosylase 1, was previously reported to protect against mitochondrial DNA (mtDNA) damage and ventilator induced lung injury (VILI). In the present study we determined whether mitochondrial targeted endonuclease III (EndoIII) which cleaves oxidized pyrimidines rather than purines from damaged DNA would also protect the lung. Minimal injury from 1 h ventilation at 40 cmH_2_O peak inflation pressure (PIP) was reversed by EndoIII pretreatment. Moderate lung injury due to ventilation for 2 h at 40 cmH_2_O PIP produced a 25-fold increase in total extravascular albumin space, a 60% increase in W/D weight ratio, and marked increases in MIP-2 and IL-6. Oxidative mtDNA damage and decreases in the total tissue glutathione (GSH) and the GSH/GSSH ratio also occurred. All of these indices of injury were attenuated by mitochondrial targeted EndoIII. Massive lung injury caused by 2 h ventilation at 50 cmH_2_O PIP was not attenuated by EndoIII pretreatment, but all untreated mice died prior to completing the two hour ventilation protocol, whereas all EndoIII-treated mice lived for the duration of ventilation. Thus, mitochondrial targeted DNA repair enzymes were protective against mild and moderate lung damage and they enhanced survival in the most severely injured group.

## 1. Introduction

There is now general acceptance that ventilator induced lung injury (VILI) contributes significantly to the mortality in the acute respiratory distress syndrome (ARDS) because a large scale clinical trial reducing tidal volume from 12 mL/kg to 6 mL/kg improved ARDS survival by 22% [1,2]. However, tidal volume cannot be further reduced because a minimal tidal volume is necessary to preserve life. Unfortunately even using a low tidal volume, mechanical ventilation can magnify the injurious effect of pre-existing damage or infection [3,4,5]. This means a further improvement in survival from ARDS during mechanical ventilation will require a pharmacologic intervention which will protect against injury due to a variety of insults that may activate multiple signal pathways. There is increasing evidence that impairment of mitochondrial function by excess oxidant injury may play a central role in lung injury secondary to mechanical stress as well as septic challenge. 

Although mitochondria produce superoxide during normal energy production, cyclical stretch of lung endothelial and epithelial cells produces an excess of oxidants that can damage mitochondrial proteins and DNA. In addition, mitochondrial DNA (mtDNA) is some 10 to 100-fold more sensitive to oxidative damage than nuclear DNA [6]. These damaged mitochondria can leak increased amounts of superoxide into the cytoplasm and activate NADPH oxidases [7,8]. A vicious positive feedback of oxidants between mitochondria and NADPH oxidases can then cause progressive mtDNA damage and loss of mitochondrial function [6,9,10]. Repair of mtDNA damage can interrupt the progression of oxidant production, mitochondrial failure and cell death.

Previously, oxidant challenged pulmonary endothelial cells and other cell types pretreated with the mitochondrial targeted DNA repair enzymes, 8-oxoguanine glycosylase (OGG1) and Endonuclease III (EndoIII), had significantly reduced mtDNA oxidative damage, apoptosis, and cell death [11,12,13]. In a recent study, mitochondrial targeted OGG1 attenuated the acute increases in lung permeability and inflammation in lungs of intact mice ventilated with high peak inflation pressures (PIP) [14]. Since the activity of OGG1 repairs oxidant damaged purines, we investigated whether EndoIII, which repairs oxidized pyrimidines, would have the same protective effect in intact animals. We found that EndoIII had similar beneficial effects on lung permeability, inflammation and survival in high PIP ventilated mice as did OGG1 treatment with only minor quantitative differences.

## 2. Experimental Section 

All experimental protocols were approved by the Institutional Animal Care and Use Committee of the University of South Alabama, College of Medicine. Anesthetized mice were euthanized by exsanguination at the time of flushing blood out from the lung.

### 2.1. Fusion Protein Constructs 

Codon optimized constructs were placed in plasmids for expression in *E. coli* of fusion proteins containing EndoIII coupled to a TAT sequence to facilitate cellular uptake, the MTS from MnSOD, a hemaglutin (HA) tag for immunological localization and a histidine tail as previously described [15]. Liquid cultures of bacterial cells transfected with plasmids containing the constructs were grown to an OD60 = 0.6 and induced with IPTG for 3 h. Bacteria were pelleted by centrifugation and resuspended in buffer A (20 mM Tris-HCl pH 8.0, 500 mM NaCl, 1 × protein inhibitor cocktail EDTA-free (EMD Millipore, Billerica, MA, USA), 100 mM PMSF and 5 mM imidazole). Bacteria were lysed by sonication with a Branson Sonifier 250. After sonication, bacterial lysates were spun in a Beckman Ultracentrifuge for 20 min at 105 × *g*. After centrifugation, cleared lysates were incubated with Ni-NTA-agarose. The Ni-NTA-agarose was placed in a column and washed with several volumes of wash buffer (Buffer A containing 30 mM imidazole). The bound protein was eluted from the column with elution buffer (buffer A containing 500 mM imidazole) and purity of the eluted protein was assessed using SDS-PAGE. All reagents for fusion protein production were obtained from Sigma-Aldrich (St. Louis, MO, USA) unless otherwise indicated. Previous studies have established that the EndoIII fusion construct localizes almost exclusively in the mitochondrial subcellular fraction with little or no detectable accumulation in nucleus or cytosol [16].

### 2.2. Treatment with mt-Targeted EndoIII

Approximately 24 h before ventilation, C57BL/6 male mice (Charles River, Wilmington, MA, USA), weighing 20.2–41.9 g (25.7 ± 4.0 g), were anesthetized with an intraperitoneal injection of Ketamine (90 mg/kg) and pentobarbital sodium (25 mg/kg). The left jugular vein was exposed, and the mice infused I.V. with fusion protein constructs containing EndoIII (70 µg) diluted in PBS to 30 μL. Untreated mice were injected with PBS only. After bleeding stopped, the incision was sutured and the mice were allowed to recover.

### 2.3. Experimental Protocols

Ventilation protocols were designed to produce three levels of severity of lung injury ranging from minimal to very severe. Mice were anesthetized with an intraperitoneal injection of ketamine (90 mg/kg) and pentobarbital sodium (25 mg/kg). The trachea was cannulated, and the mice were ventilated with 100% oxygen using a Harvard rodent ventilator (model No. 683: Harvard, South Natick, MA, USA). Mice received either no ventilation (No Vent., n = 5), ventilation for 1 h with either 10 cmH_2_O PIP (PIP10 × 1 h Vent., n = 5), 40 cmH_2_O PIP ventilation only (PIP40 × 1 h Vent., n = 5), 40 cmH_2_O PIP ventilation with EndoIII (PIP40 × 1 h + EndoIII, n = 5), 2 h ventilation with 40 cmH_2_O PIP only (PIP40 × 2 h Vent., n = 5), 2 h ventilation with 40 cmH_2_O PIP after EndoIII (PIP40 × 2 h + EndoIII, n = 6), 2 h ventilation with 50 cmH_2_O PIP only (PIP50 × 2 h Vent., n = 5), or 2 h ventilation with 50 cmH_2_O PIP after EndoIII (PIP50 × 2 h + EndoIII, n = 5). The approximate tidal volumes used were 0.3 mL (12 mL/kg) for the 10 cmH_2_O PIP group; 0.8 mL (32 mL/kg) for the 40 cmH_2_O PIP groups, and 0.95 mL (36 mL/kg) for the 50 cmH_2_O PIP groups [17,18]. After the ventilation period, mice were injected with 50 IU heparin into the peritoneal space, blood was collected by cardiac puncture of the left ventricle and blood gases were determined using a Radiometer America (Westlake, OH, USA) ABL5 blood gas machine. Ventilation rates were decreased during high PIP ventilation compared to low PIP ventilation groups. However, this reduction was not sufficient to prevent some degree of hyperventilation and hypocapnia in the high PIP ventilation groups. A suture was placed around the pulmonary artery and aorta and a cannula (0.86 mm ID, 1.27 mm OD) placed in the pulmonary artery. The hilum of the right lung was tied off and the left ventricle was clipped. The left lung was flushed of blood with 2 mL of 10% phosphate-buffered saline (PBS), and bronchoalveolar lavage (BAL) was performed 2 times with 0.3 mL of saline on the left lung. After BAL, the left lung was harvested, minced and sonicated using a Missonex XL 2000 Sonicator (Farmingdale, NY, USA) in 3 second bursts with 0.5 mL 10% PBS. After centrifugation to obtain the supernatant, the pellet dried to a constant weight for tissue dry weight. Collected blood was centrifuged and serum was separated.

### 2.4. Western Immuno-Blot Analysis of Sub-Cellular Fusion Protein Localization

Sub-cellular fractions were prepared from lung homogenates as described previously [16]. Lung tissue (1 g) was homogenized in a glass homogenizer with a Teflon pestle eight times using 6 mL of homogenization buffer (0.25 M sucrose, 20 mM Hepes-NaOH pH 7.4, and 1 mM EDTA). Protease inhibitor cocktail (Sigma-Aldrich) was added to all isolation buffers. The homogenate was filtered through 70 μm mesh (BD Biosciences, Bedford, MA, USA) and centrifuged on a cushion (5 mL) containing 0.35 M sucrose, 20 mM Hepes-NaOH pH 7.4, and 1 mM EDTA at 700 g for 10 min at 4 °C. The fraction around and above the interphase was collected as crude mitochondria and reserved for mitochondrial isolation. The nuclear pellet was suspended in 3 mL of nuclear isolation buffer (0.25 M sucrose, 20 mM Hepes-NaOH pH 7.4, 25 mM KCl and 5 mM MgCl2) and purified on 3 mL cushion containing 0.8 M sucrose, 20 mM Hepes-NaOH pH 7.4, 25 mM KCl and 5 mM MgCl_2_ at 3,000 *g* for 15 min at 4 °C. The nuclear pellet so obtained was washed with nuclear isolation buffer and centrifuged at 1,000 *g* for 10 min. The pellet containing purified nuclei was suspended in 300 μL of RIPA buffer (Cell Signaling Technology, Danvers, MA, USA), incubated for 30 min on ice, and centrifuged at 18,000 *g* for 15 min. The supernatant was designated as the “nuclear fraction.” The crude mitochondrial fraction, collected as described above, was centrifuged at 18,000 *g* for 20 min to pellet mitochondria, which were suspended in 2 mL of mitochondrial isolation buffer (0.2 M mannitol, 50 mM sucrose, 20 mM Hepes-NaOH pH 7.4, and 1 mM EDTA) and centrifuged under the same conditions. This supernatant was designated as the cytosolic fraction, while the pellet containing mitochondria was suspended in 300 μL of RIPA buffer (Cell Signaling Technology), incubated for 30 min on ice and centrifuged at 18,000 *g* for 15 min. This latter supernatant was designated as the mitochondrial fraction. Cytosolic, nuclear and mitochondrial fractions were subjected to Western immunoblot analysis for specific markers and for HA-tagged fusion protein constructs.

Western blot analyses were performed as described earlier using antibodies against the HA tag (Sigma-Aldrich) to determine sub-cellular distribution of the fusion proteins [16]. The mitochondrial fraction was characterized using an antibody (Sigma-Aldrich) against the cytoplasmic loop of the voltage-dependent anion channel (VDAC, porin-1, Sigma-Aldrich), the pore forming unit in the outer mitochondrial membrane which serves as an adenine nucleotide translocator. The nuclear fraction was characterized using an antibody against Lamin B1 (Santa Cruz Biotechnology, Santa Cruz, CA, USA) a component of the nuclear envelope. An antibody against β-actin was used as a loading control for total lysate and cytosolic fractions.

### 2.5. Measurement of Albumin Plasma Equivalents

Albumin quantities in BAL, supernatant of homogenized left lung tissue and serum were measured by using an ELISA kit (Bethyl Labs, Montgomery, TX, USA) for mouse albumin. The left lung was minced and sonicated in 500 µL PBS using a Missonex XL 2000 sonicator. Supernatant samples were removed and the left lung tissue desiccated at 80 °C for 5 days to obtain a stable dry weight. Initial dilutions for ELISA were: 1 × 10^6^ for serum, 4 × 10^3^ for BAL, and 6 × 10^3^ for tissue supernatant. Serum samples were further diluted by 1:3, then 200 µL of diluted serum, BAL and tissue supernatant samples were each spotted and followed by three successive 1:2 dilutions. Four wells each of PBS blank and positive albumin controls in the sample concentration range were also included. Then 100 µL of anti-albumin detection antibody was added to each well and the plate incubated for 1 hour at room temperature on an orbital shaker. Contents were discarded and the plate washed 4 times. Next, 100 µL of horseradish peroxidase solution was added and the plate incubated for 30 min at room temperature on an orbital shaker. This was followed by 100 µL of colorimetric substrate incubated for 30 min followed by 100 µL of the stop solution supplied in the ELISA kit. The resultant yellow color was read at 450 nm with a Dynex MXR plate reader (Dynex Technologies, Chantilly, VA, USA). Sample albumin concentrations were calculated from each serial dilution and checked for consistency. Mouse albumin standard was mixed with PBS to obtain an initial concentration of 0.9 µg/mL. Two standard curves were determined for each plate using seven 1:3 dilutions with final volumes of 200 µL per well and expressed using a four parameter curve fit. Details of plate layouts are shown in an online supplement. Residual albumin in the tissue supernatant was assumed to represent primarily interstitial albumin and alveolar compartment albumin was assumed to be largely removed by the BAL procedure. Total albumin masses (QA) were calculated for tissue (QA,tiss), alveolar spaces (QA,alv) and total extravascular albumin (QA,total = QA,tiss + QA,alv). These were normalized to left lung tissue dry weight (DLW) and plasma albumin concentration (CA,pl) and expressed as plasma equivalents (PE) in microliters/micrograms, where:

PE = QA/CA,pl/DLW (1)

Use of this normalization technique corrects for differences in plasma albumin concentration and lung weights between experiments. The sensitivity of this kit was 20 ng/mL. Lung vascular injury was evaluated using increases in the total, BAL, and tissue endogenous albumin plasma equivalents in each group.

### 2.6. Lung Wet-To-Dry Weight Ratios

The right lower lobe was weighed (W) and desiccated at 80 °C for 1 week to obtain a stable dry weight (D) for calculation of the wet-to-dry weight ratio (W/D ratio).

### 2.7. Measurement of MIP-2 and IL-6

MIP-2 and IL-6 in bronchoalveolar lavage fluid (BALF) were measured using mouse ELISA kits (R&D Systems). The sensitivity of these kits were 1 pg/mL for MIP-2 and 2.5 pg/mL for IL-6.

### 2.8. Analysis of mtDNA Content and Oxidative Damage

Immediately after perfusion, lungs were snap-frozen in liquid nitrogen and saved for determination of oxidative mtDNA damage. Total DNA was isolated from lung samples and powdered with a mortar and pestle using previously described methods [19,20]. Purified DNA samples were digested with PpuMI and AhdI restriction enzymes (New England Biolabs) and used for further analyses.

To measure oxidative damage to the mitochondrial genome, a quantitative Southern blot analysis was performed. In brief, digested DNA samples were precipitated, dissolved in TE buffer and precisely quantified on the Hoefer DyNA Quant 200 Fluorometer (Hoefer, San Francisco, CA, USA) using Hoechst 33258 dye. To reveal oxidative base modifications, DNA was treated with formamidopyrimidine glycosylase (Fpg, New England Biolabs), a bacterial DNA repair enzyme that cleaves DNA at sites of oxidized purines, thereby creating single-strand breaks. Subsequently, Fpg-treated and untreated samples were incubated with 0.1 N NaOH for 15 min at 37 °C, mixed with loading dye and resolved on 0.6% agarose alkaline gel. After electrophoresis, DNA was vacuum transferred to a nylon membrane (Roche Diagnostics, Mannheim, Germany) and hybridized with a PCR-generated probe to the corresponding region of mtDNA. The mtDNA probe, labeled with a DIG-labeling kit (Roche Diagnostics), was generated using rat mtDNA sequence as template and the following primers: 5′-CCCTACTTACTGGCTTCAATCTAC-3′ for the sense strand and 5′-CATACCATACCTATATATCCGAAGG-3′ for the anti-sense strand. The 1016- bp product was hybridized with a 13.6-kb fragment of rat mtDNA obtained after PpuMI and AhdI digestion*.* Hybridization bands were detected with Amersham Hyperfilm ECL (GE Healthcare, Piscataway, NJ, USA) and a Gel Logic 1500 Imaging System (Kodak, Rochester, NY, USA). Changes in the equilibrium lesion density of Fpg-detectable base oxidation lesions within each experimental group were calculated as negative *ln* of the quotient of hybridization intensities in Fpg-treated and non-Fpg bands and normalized to 10 kb (3) and are independent of the total amount of mtDNA.

### 2.9. Statistical Analysis

All values are expressed as mean ± SE. One-way analysis of variance (ANOVA) with repeated measures followed by a Student-Newman-Keuls post-test was used. Significant differences were determined where *p* < 0.05.

## 3. Results and Discussion

### 3.1. Ventilation Parameters and Arterial Blood Gasses

Respiratory rate was reduced in the groups ventilated with higher PIP levels to maintain minute ventilation near normal levels, but hypocapnia was present in the high PIP ventilation groups indicating a level of hyperventilation. Table 1 shows the ventilation times, rates and peak inflation pressures (PIP) as well as blood gases obtained from the left ventricle at the end of the experiments. PaCO_2_ values were significantly lower in the EndoIII treated groups ventilated at 40 and 50 cmH_2_O PIP, compared to non-ventilated mice although a significant alkalosis was not present. PaO_2_ values were significantly greater in the 40 cmH_2_O PIP groups ventilated for 1 h compared to the non-ventilated group. 

**Table 1:** Ventilation parameters and arterial blood gasses for all groups.

Treatment	PIP (cmH2O)	Vent. Time (min)	PaO_2_ (mmHg)	PaCO_2_ (mmHg)	pH	Vent. Rate Breaths/min
NV	10	11.2 ± 1.2	211 ± 11	36.0 ± 1.2	7.28 ± 0.01	110
Low PIP	10	60	293 ± 7	34.5 ± 2.4	7.33 ± 0.01	110 ± 2
PIP40	40	60	466 ± 14*	27.6 ± 1.1	7.35 ± 0.01	28.3 ± 0.5
PIP40 EndoIII	40	60	457 ± 12*	22.0 ± 1.6*	7.40 ± 0.02	30.4 ± O.2
PIP40	40	120	344 ± 4	26.3 ± 0.5	7.36 ± 0.01	28.3 ± 0.3
PIP40 EndoIII	40	120	478 ± 5*	21.7 ± 1.0**	7.35 ± 0.04	31.8 ± 0.2
PIP50	50	96.6 ± 7	NM	NM	NM	25.6 ± 0.2
PIP50 EndoIII	50	120	335 ± 11	17.2 ± 1.3**	7.29 ± 0.08	26.6 ± 1.0

*p < 0.05 *vs.* NV group; **p < 0.05 *vs.* NV and Low PIP group. Vent. = ventilation; PIP = peak inflation pressure.

### 3.2. Subcellular Localization of the Fusion Protein

**Figure 1:**
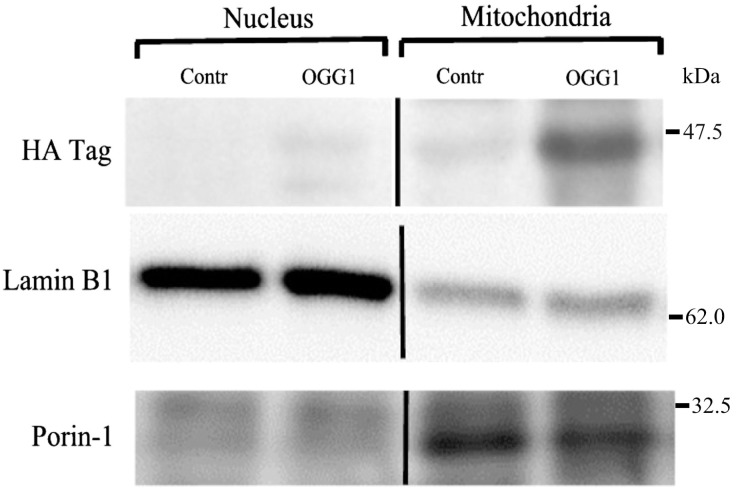
Immunoblot probed for the hemaglutin (HA) tag of the fusion protein in nuclear and mitochondrial subcellular fractions of lungs from non-ventilated control mice (Contr) and mice injected with the fusion protein construct. The nuclear protein, lamin B1, and the mitochondrial protein, porin-1, are labeled.

Injected fusion proteins rapidly localized in lung mitochondria. Figure 1 shows a representative immunoblot of the HA-tagged fusion protein in the nuclear and mitochondrial fractions of lung homogenates in control mice and mice 1 h after injection with the fusion protein construct. 

Also indicated are the nuclear envelope protein, lamin B1, and the mitochondrial outer membrane protein, porin-1, in the respective subcellular fractions. The HA tag was not detected in the nuclear fraction but was present in the mitochondrial fraction. Some slight non-specific antibody binding also occurred in the control mice but was far less prominent than in the fusion-protein injected mice. 

### 3.3. Lung Extravascular Albumin Spaces 

**Figure 2:**
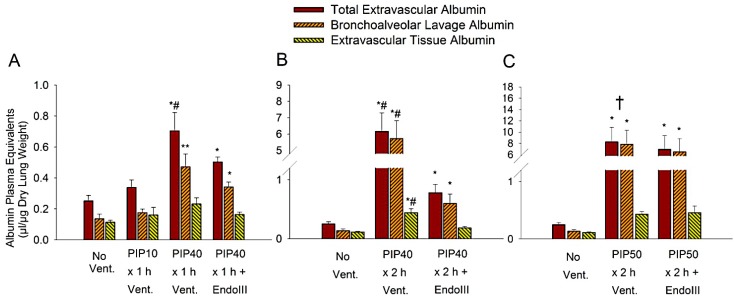
Albumin extravascular plasma equivalent spaces in mouse lung after ventilation for different times and with different peak inspiratory pressures (PIP). Shown are total extravascular albumin space, alveolar (BAL) albumin space, and interstitial (extravascular tissue albumin) space. (**A**) Mice received either No Ventilation (No Vent.), or 1 h of ventilation with 10 cmH2O PIP (PIP10), or 40 cmH2O PIP only (PIP40), or 40 cmH2O PIP with EndoIII (PIP40 EndoIII). (**B**) Mice received either no Ventilation (No Vent.), or 2 h of ventilation with 40 cmH2O PIP only (PIP40), or 40 cmH2O PIP with EndoIII (PIP40 EndoIII). (**C**) Mice received either no Ventilation (No Vent.), or 2 h of ventilation with 50 cmH2O PIP only (PIP50), or 50 cmH2O PIP with EndoIII (PIP50 EndoIII). *p < 0.05* vs.* No Vent. group. **p < 0.05* vs.* No Vent. group and PIP10 groups. #*p* < 0.05* vs.* all other groups. † All animals in the untreated PIP50 group died prior to 2 h.

The accumulation of access extravascular albumen in lung alveolar fluid and tissue were used as an index of vascular permeability. High PIP ventilation increased albumin leak from the lung capillaries in proportion to the distending pressure and the exposure time to that pressure,* i.e.*, the ventilation time. Alveolar albumen indicates albumen which has leaked across both endothelial and epithelial layers, whereas interstitial albumin represents albumen which has leaked across the endothelium but is retained in the lung tissue. Figure 2 shows the total lung, alveolar, and interstitial equivalent albumin spaces normalized to plasma concentration for the three ventilation protocols. 

Figure 2A shows the least injurious protocols consisting of one hour of ventilation with either 10 cmH_2_O PIP or 40 cmH_2_O PIP. In untreated animals, total extravascular albumin spaces increased significantly after 1 h of 40 cmH_2_O PIP by 2.8 -fold compared to unventilated lungs, and 2.2-fold compared to the 1 h 10 cmH_2_O PIP ventilation group, and was significantly higher than that of other groups. In the EndoIII treated group, total extravascular albumin spaces increased significantly by some 2.1-fold compared to unventilated mice, but this increase was significantly less than that of the untreated 40 cmH_2_O PIP ventilation group. The alveolar albumin space was significantly increased in both groups ventilated with 40 cmH_2_O PIP compared to the groups receiving no ventilation, or ventilation with only 10 cmH_2_O PIP. There were no differences in the interstitial albumin spaces. Figure 2B indicates dramatic increases in lung albumin spaces after ventilation with 2 h of 40 cmH_2_O PIP compared to unventilated mouse lungs. Total extravascular albumin space increased 25-fold, alveolar extravascular albumin space increased 42-fold and interstitial extravascular albumin space increased 3.7-fold in untreated mice compared to lungs of non-ventilated mice. However, lung total, alveolar and interstitial extravascular albumin spaces increased by only approximately 2.5-fold in the EndoIII treated lungs compared to non-ventilated lungs, and these values were significantly less than values in the untreated group ventilated at 40 cmH_2_O PIP. Figure 2C shows lung extravascular albumin spaces after two h ventilation at 50 cmH_2_O PIP. Very large increases in lung total, alveolar and interstitial extravascular albumin spaces occurred in both the untreated and treated groups at this high level of PIP ventilation when compared to nonventilated lungs. These increases were highly significant but there were no statistical differences in albumin spaces between the untreated and EndoIII treated groups. However, all of the mice in the untreated 50 cmH_2_O PIP groups died prior to completing the two-hour ventilation protocol, whereas all mice in the EndoIII treated group survived the protocol.

### 3.4. Lung Wet/Dry Weight Ratios

**Figure 3:**
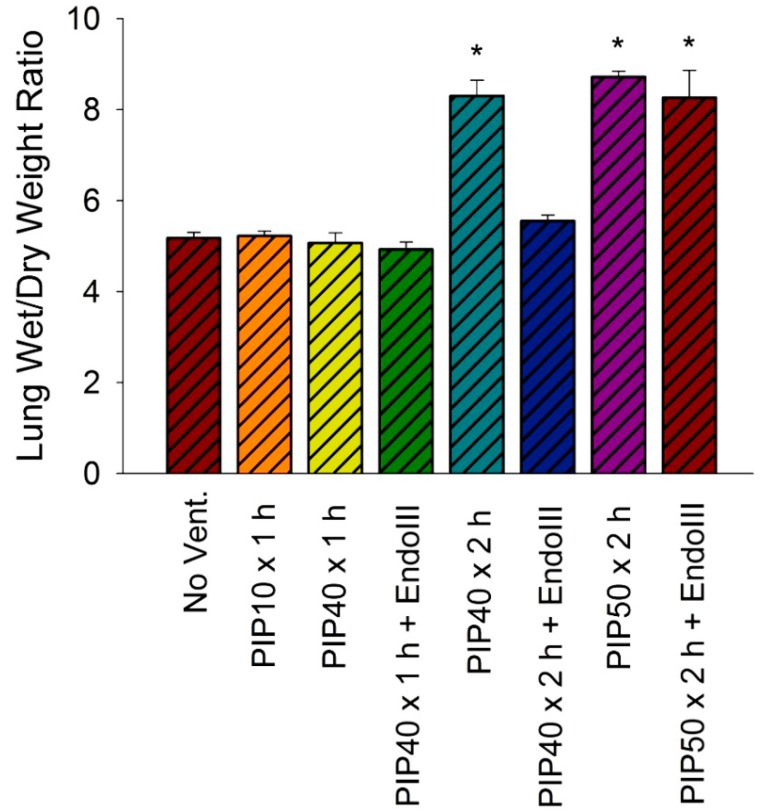
Lung wet/dry weight ratios for all experimental groups. *p < 0.05* vs.* No Vent., PIP10 × 1 h Vent. only, PIP40 × 1 h Vent. only, PIP40 × 1 h + EndoIII, and PIP40 × 2 h + EndoIII groups.

The lung wet to dry weight ratio indicates the edema accumulation in the lung and is a function of the rate of fluid filtration across the lung capillaries. Figure 3 summarizes the lung wet/dry weight ratios as an indicator of lung edema in all experimental groups. There were no significant differences in lung wet/dry weight ratios between the groups receiving no ventilation, 1 h ventilation with 10 cmH_2_O PIP or 40 cmH_2_O PIP without treatment, or the group ventilated for either 1 or 2 h at 40 cmH_2_O PIP pretreated with EndoIII. However, 2 h of ventilation with 40 cmH_2_O PIP without EndoIII pretreatment resulted in a significant 60% increase in lung wet/dry weight ratios, as did ventilation for 2 h at 50 cmH_2_O PIP in both untreated and EndoIII treated mice. There were no significant differences between the two groups ventilated with 50 cmH_2_O PIP and the untreated group ventilated for 2 h at 40 cmH_2_O PIP.

### 3.5. Bronchoalveolar Lavage Inflammatory Cytokines

Inflammatory cytokines were measured in the bronchoalveolar lavage (BAL) fluid as an indicator of inflammation during mechanical injury. Interleukin-6 (IL-6) and macrophages inflammatory protein-2 (MIP-2) levels are correlated with inflammation and injury [21]. Both cytokines are secreted by macrophages, MIP-2 is chemotactic for neutrophils, and IL-6 has both pro-and anti-inflammatory actions. These cytokines exhibited increases that were proportional to ventilation times and peak airway pressures and mirrored the degree of albumen accumulation and the protective effect of the mitochondrial DNA repair enzyme. 

**Figure 4:**
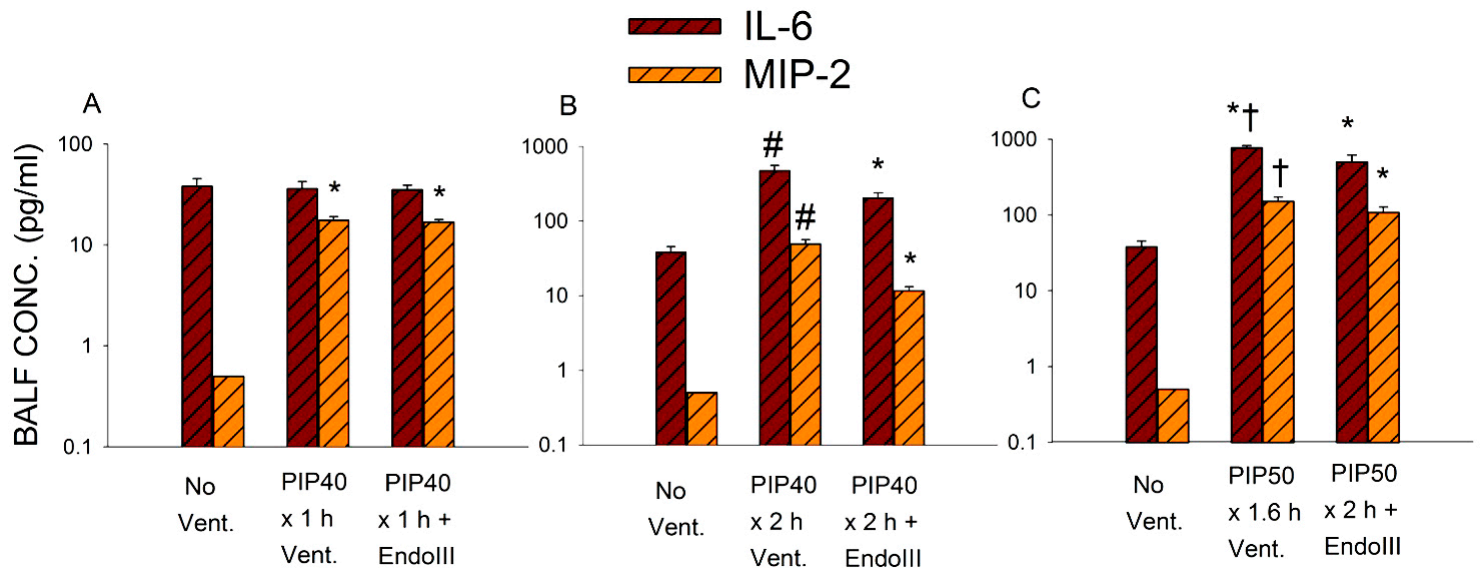
Interleukin-6 (IL-6) and macrophage inflammatory protein-2 (MIP-2) concentrations in bronchoalveolar lavage (BAL) fluid obtained from lungs of mice in all ventilation protocols shown on log scales. (**4A**) Groups received either no ventilation (No Vent.), or ventilation for 1 hour without treatment at 10 cmH_2_O PIP (PIP10) or ventilation for 1 hour with 40 cmH_2_O PIP (PIP40) with and without pretreatment with EndoIII. (**4B**) Groups received either no ventilation (No Vent.), or ventilation for 2 h with 40 cmH_2_O PIP (PIP40) with and without pretreatment with EndoIII. (**4C**) Groups received either no ventilation (No Vent.), or ventilation for 2 h with 50 cmH_2_O PIP (PIP50) with and without pretreatment with EndoIII. *p < 0.05* vs.* No Vent. group. #*p* < 0.05* vs.* all other groups. † All animals in the untreated PIP50 group died prior to 2 h.

Figure 4 summarizes the effect of the three levels of lung injury on BAL IL-6 and MIP-2 concentrations. Note that these Y axis scales are logarithmic and that the range for 4B and 4C is 10 times that of 4A. Groups ventilated for one hour at 40 cmH_2_O PIP showed a small but significant increase in BAL MIP-2 but no change in IL-6 compared to non-ventilated values. Figure 4B indicates that both IL-6 and MIP-2 values increased significantly in all mice ventilated for 2 h at 40 cmH_2_O PIP compared to non-ventilated controls. Although cytokine levels were significantly increased in mice treated with EndoIII compared to non-ventilated controls, these levels were significantly reduced compared to untreated mice. As shown in 4C, BAL IL-6 and MIP-2 reached the highest levels after 2 h ventilation with 50 cmH_2_O PIP, but there were no significant differences between IL-6 or MIP-2 values in EndoIII treated and untreated mice. However, all untreated mice died prior to completing the two hour ventilation protocol, whereas all EndoIII treated mice survived the entire 2 h.

### 3.6. Survival During 2 h Ventilation at 50 cmH_2_O PIP

**Figure 5:**
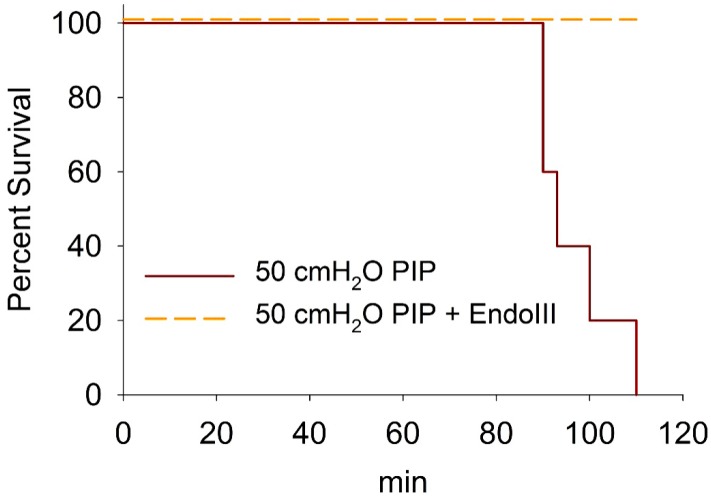
Kaplan-Meier survival plot for animals ventilated at 50 cmH2O PIP for 2 h showing EndoIII treated (dashed line) and untreated (solid line) groups.

A Kaplan-Meier survival plot for animals ventilated at 50 cmH_2_O PIP is shown in Figure 5. Approximately half the untreated animals died within 1 ½ h after starting ventilation with 50 cmH_2_O PIP, whereas all of the animals pretreated with EndoIII survived the 2 h of ventilation.

### 3.7. Lung Glutathione Concentrations

**Figure 6:**
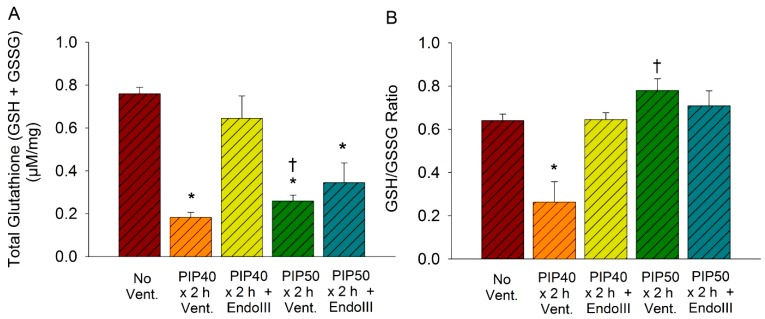
Lung tissue glutathione concentrations in groups ventilated for 2 h with 40 cmH2 O PIP, and 2 h with 50 cmH2 O PIP. Shown are (**6A**) total glutathione pool (GSH+GSSG) and (**6B**) the GSH/GSSG ratios. *p < 0.05* vs.* No Vent. group. † All animals in the untreated PIP50 group died prior to 2 h.

Lung glutathione levels were measured to evaluate the redox status of the lung. As indicated by Figure 6A, the total lung tissue glutathione pool (GSH + GSSG) was significantly reduced in untreated mice ventilated at 40 cmH_2_O or 50 cmH_2_O PIP for 2 h compared to nonventilated controls. However, pretreatment with EndoIII prevented the decrease in total GSH after 2 h ventilation with 40 cmH_2_O but not after 2 h ventilation with 50 cmH_2_O. There was no significant difference between the EndoIII treated group ventilated for 2 h at 40 cmH_2_O and the nonventilated control group. Figure 6B indicates that the GSH/GSSG ratio was also significantly reduced in the group ventilated for 2 h at 40 cmH_2_O PIP compared to nonventilated controls, but EndoIII prevented the decrease caused by ventilation with 2 h at 40 cmH_2_O PIP treated group and this value was not significantly different from the non-ventilated group. However, the GSH/GSSG ratios did not significantly decrease in either the treated and untreated groups ventilated 2 h with 50 cmH_2_O PIP, as these values were not significantly different from the nonventilated controls. Thus, EndoIII treatment preserved the glutathione antioxidant system after ventilation for 2 h with 40 cmH_2_O PIP but not after ventilation for 2 h at 50 cmH_2_O PIP.

### 3.8. Lung Mitochondrial DNA Damage

**Figure 7:**
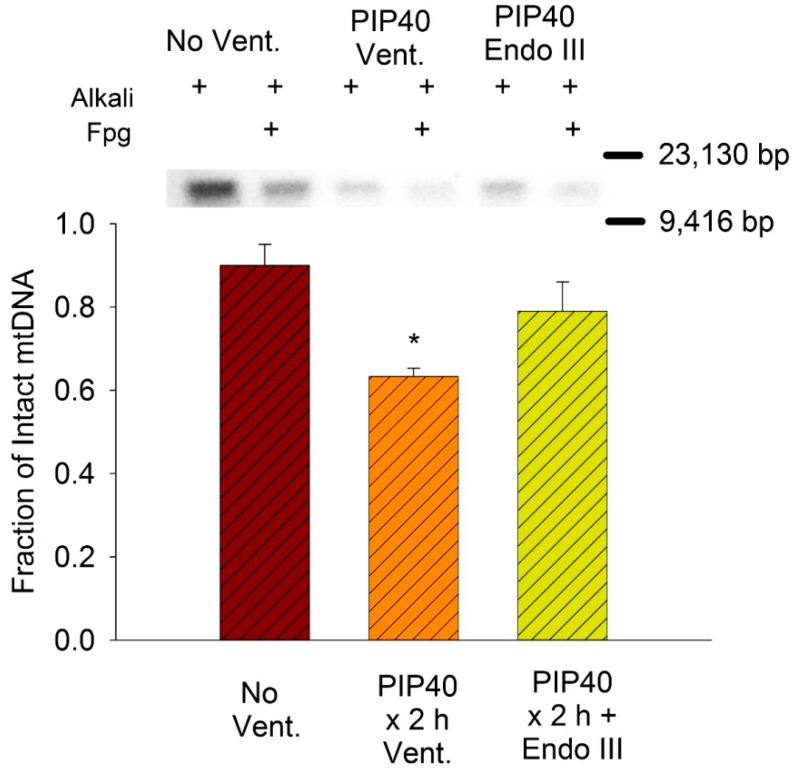
Southern blot analysis of mitochondrial DNA in lung tissue in groups ventilated for 2 h with 40 cmH2O PIP. Samples were treated with alkali to reveal mtDNA strand breaks and alkali + Fpg to indicate base damage. Bar graph summarizes mean fractions of intact* vs.* total mitochondrial DNA in lungs of non-ventilated controls (No Vent.), and ventilated mice either untreated (PIP40 × 2 h Vent.) or EndoIII treated (PIP40 × 2 h + EndoIII) after ventilation for 2 h at 40 cmH2O PIP. *p < 0.05* vs.* No Vent. group.

The amount of mtDNA damage was measured in the non-ventilated lungs and lungs of EndoIII treated and untreated groups ventilated for 2 h at 40 cmH_2_O PIP. Groups in this ventilation protocol were selected because they demonstrated the greatest differential effect of EndoIII on injury. Figure 7 summarizes the Southern blot analysis of Fpg-detectable oxidative base damage. Only the non-ventilated group and groups ventilated for 2 h at 40 cmH_2_O PIP were analyzed because these groups demonstrated the greatest differential effect of the targeted mtDNA repair enzymes on injury. The fraction of intact mitochondrial DNA (13,065 bp) after Fpg treatment in lungs ventilated at 40 cmH_2_O PIP was significantly reduced in untreated lungs compared to non-ventilated controls and mice treated with mitochondrial-targeted EndoIII. 

## 4. Discussion

During the process of oxidative phosphorylation, mitochondria leak superoxide anions from the electron transport pathway at Complex 1, 2 and 3 [7]. Although superoxide generated is converted to H_2_O_2_ by manganese superoxide dismutase, the GSH/GSSG ratio is lower in mitochondria compared to that in the cytoplasm [22]. Excessive oxidant stress can overwhelm the antioxidants and damage mitochondrial DNA, and the mitochondrial DNA is some 10 to 100 times more susceptible to oxidative damage than nuclear DNA [10]. Damage to mtDNA can result in a vicious cycle of positive feedback whereby mtDNA damage produces more superoxide, resulting in activation of NADPH oxidases, progressively more mtDNA damage with ultimate mitochondrial failure and cell death [7,9]. Oxidative mtDNA damage under baseline conditions is rapidly repaired through the base excision repair (BER) pathway [23]. Oxidized purines are excised by 8-oxoguanine DNA glycosylase (OGG1) and oxidized pyrimidines by endonuclease III-like protein 1 in mammalian cells, or endonucleases in bacteria. After base removal by these glycosylases, the abasic sites are processed by Ref-1/APE1 and a new base inserted. This is followed by re-attachment of the cut strands by a DNA ligase [23]. 

In previous studies of oxidant challenged pulmonary endothelial cells and other cell types, pretreatment with the mitochondrial targeted DNA repair enzymes, 8-oxoguanine glycosylase (OGG1) and Endonuclease III (EndoIII) significantly reduced mtDNA oxidative damage, apoptosis, and cell death [11,12,13]. These repair enzymes were targeted to mitochondria by a fusion protein which consists of the TAT sequence from HIV to facilitate cellular uptake, the mitochondrial targeting sequence from Mn-SOD, and either of the DNA repair enzymes, OGG1 or EndoIII [12,24]. More recent studies demonstrated a protective effect of mitochondrial targeted repair enzymes against oxidant induced lung injury caused by direct oxidant stress [16], high peak inflation pressures [14] and intra-tracheal *Pseudomonas aeruginosa* [25].

In our previous VILI study, we demonstrated that OGG1 pretreatment protected against VILI and death in intact mice [14]. We show here that EndoIII, which replaces oxidized pyrimidines rather than oxidized purines, had an almost identical protective effect against mechanical lung injury. There were small quantitative differences between OGG1 and EndoIII in the measured variables. In particular, the BAL cytokine levels in EndoIII treated animals were slightly greater than observed in OGG1 treated animals but still dramatically lower than those in untreated animals ventilated for 2 h with 40 cmH_2_O PIP. The present study shows that the fusion protein localized in the mitochondria of lung tissue and significantly protected against mechanical induced microvascular protein leakage. During minimal lung damage (40 cmH_2_O PIP × 1 h), an increased extravascular lung albumin was detected even though the wet/dry weight ratio did not increase significantly. The small extravascular albumin increase was significantly reduced after EndoIII treatment. The most dramatic protection occurred with moderately severe vascular injury (40 cmH_2_O PIP × 2 h), where the 25-fold increase in extravascular albumin was almost completely prevented by EndoIII treatment. As with OGG1 treatment, pretreatment with EndoIII did not significantly protect against devastating lung injury during ventilation with 50 cmH_2_O PIP for 2 h. However, as was observed with OGG1 treatment, all of the EndoIII treated animals survived the 2 h ventilation at 50 cmH_2_O PIP, whereas none of the untreated animals survived. 

Similar to OGG1 treatment, the EndoIII significantly protected against the increase in inflammatory cytokines, depletion of lung tissue GSH, the decrease in the GSH/GSSG ratio and the increase in mtDNA degradation [14]. These results indicate a significant attenuation of the overall inflammatory response to mechanical injury and have significant implications for the clinical intensive care management of patients with ARDS. Although reduction of tidal volumes during mechanical ventilation has significantly improved survival, current tidal volume settings are minimal and cannot be further reduced [1,26]. This necessitates a pharmacologic approach to further preserve life during this critical injury. The improved survival offered by EndoIII or OGG1 pretreatment also has implications for the multiple organ failure syndrome (MODS) which persists as a major source of fatalities in these patients, with a mortality rate of almost 70% [27]. Since the EndoIII fusion protein was injected systemically, we can assume that the targeted enzymes reached the mitochondria of other organs. Preservation of cardiac output and other vital functions may then have contributed to the increased survival, although EndoIII deposition in peripheral organs or organ function were not measured in this study. However, mitochondrial targeted antioxidants were recently shown to protect against systemic inflammation and MODS [28].

Although it is clear that supplementation of mitochondrial repair enzymes has a dramatic protective effect against a variety of insults that induce oxidant stress, the mechanism of protection is unclear. Lung vascular injury was accompanied by an increased oxidative base damage in the mitochondrial genome, and involvement of reactive oxygen and nitrogen species in VILI is well documented [29]. Rapid production of ROS in response to cyclical stretch has been demonstrated for both endothelial cells and lung epithelial cells, with mitochondrial ROS generated in endothelium within min [30,31]. One possible mechanism of injury is that mtDNA damage impairs mtDNA transcription and results in deficiencies in oxidative phosphorylation proteins. This could result in increased ROS production causing a progressive positive feed-back cycle of damage responsible for cell death and dysfunction [7,9]. However, the protective effect in the intact lung experiments was much more rapid than protein transcription and similar whether purine or pyrimidine repair enzymes were supplemented in mitochondria. Other possible explanations are that there is a critical threshold for base damage and that a reduction in the critical number of either type of oxidized base prevents mitochondrial failure [24]. Still another possibility is that the ligase activities of both OGG1 and EndoIII for repair of the sugar-phosphate backbone are more significant for protection than the replacement of base lesions [24]. Additional mechanisms of protection may result from direct signaling pathways which may be rapidly activated independently of the base repair activities of these enzymes. Direct links of the mtDNA complex to the cytoskeleton via trans-mitochondrial membrane spanning non-muscle myosin and actin filaments may directly affect vascular permeability [32]. Previous studies showed that inhibition of non-muscle myosin light chain kinase attenuated increases in lung vascular permeability resulting from a variety of insults, including VILI [33,34,35]. Other protective mechanisms include activation of RAS family GTPases with phosphorylation of the mitogen activated kinases, MEK1,2/ERK 1,2 [36]. Phosphatidylinositol 3-kinases also have RAS interaction sites, and pharmacologic inhibition or genetic deletion of phosphatidylinositol 3-kinase markedly attenuates VILI [37,38,39].

## 5. Conclusions

In summary, mitochondrial targeted EndoIII attenuated increased vascular permeability, inflammatory cytokine responses, oxidative mtDNA damage, and oxidative stress in the lungs after high PIP ventilation. In the presence of catastrophic lung damage, the EndoIII fusion protein also increased survival during high PIP ventilation. These mitochondrial-targeted DNA repair proteins could have potential clinical application during mechanical ventilation of ARDS patients to protect against VILI, as well as multiple organ failure.
